# A High-Birefringence Microfiber Sagnac-Interferometer Biosensor Based on the Vernier Effect

**DOI:** 10.3390/s18124114

**Published:** 2018-11-23

**Authors:** Xue-Zhou Wang, Qi Wang

**Affiliations:** College of Information Science and Engineering, Northeastern University, Shenyang 110819, China; wangxuezhou@stumail.neu.edu.cn

**Keywords:** microfiber, biosensor, Sagnac interferometer, Vernier effect

## Abstract

We propose a high-sensitive Sagnac-interferometer biosensor based on theVernier effect (VE) with a high-birefringence microfiber. The sensitivity enhancement is achieved by utilizing two cascaded Sagnac interferometers. One of the two interference loops consists of a panda polarization-maintaining fiber as a filter, whilst the other is comprised of high-birefringent microfiber coated Graphene oxide (GO) as a sensing channel. We theoretically analyzed the sensitivity of the sensor and verified it with experiments. The results of the simulation show that the refractive index sensitivity is more than five times that of the fiber sensor based on a single Sagnac loop. The sensitivity of the refractive index in the experiments can reach 2429 nm/refractive index unit (RIU), which is basically in accordance with the simulation. We also use electrostatic adsorption to coat GO on the surface of the sensing channel. GO is employed to adsorb bovine serum albumin (BSA) molecules to achieve the desired detection results, which has good biocompatibility and large specific surface area. The sensitivity to detect BSA can reach 9.097 nm/(mg×mL^−1^).

## 1. Introduction

Biosensing has many applications in bioengineering, medical science, and environmental science. Biosensing technology based on optical fibers has received extensive attention from researchers in recent years due to its bio-compatibility, high sensitivity, small size, immunity to electromagnetic interference, and ease in distributed measurement [1,2]. Various fiber-based measurement methods have been reported, such as surface plasmon resonance [3], long-range surface plasmon resonance [4], Bragg grating [5], Mach-Zehnder interferometers [6], and Fabry-Perot interferometers [7]. The Sagnac interferometer loop is also one of the important devices among them [8,9]. In general, a high-birefringent (Hi-Bi) fiber is connected to the output port of the fiber coupler to form a Sagnac loop. The birefringence of the fiber can produce the optical path difference between the orthogonally polarized fields, such as a D-shaped fiber [10], panda fiber [11], tapered fiber [12], or Hi-Bi photonic crystal fibers [13]. In addition, Sagnac loops with an Hi-Bi microfiber have a high sensitivity in analyte refractive index (RI) sensing because they exhibit a unique birefringence dispersion characteristic [14,15]. Moreover, through reducing the size of the microfiber and making the group birefringence close to zero, the sensitivity can be increased indefinitely [16].

In this paper, we propose a high-birefringence microfiber Sagnac-interferometer biosensor based on the Vernier effect. The sensor proposed in this paper employs a panda polarization-maintaining fiber and birefringence microfiber cascaded into two Sagnac loops to further improve the sensitivity of the sensor. The surface of the sensing channel is coated with GO by the electrostatic adsorption method. The good biocompatibility of graphene oxide and the large specific surface area are used to adsorb bovine serum albumin (BSA) molecules to achieve the desired detection effect.

## 2. Structure and Theory Modelling

**Figure 1:**
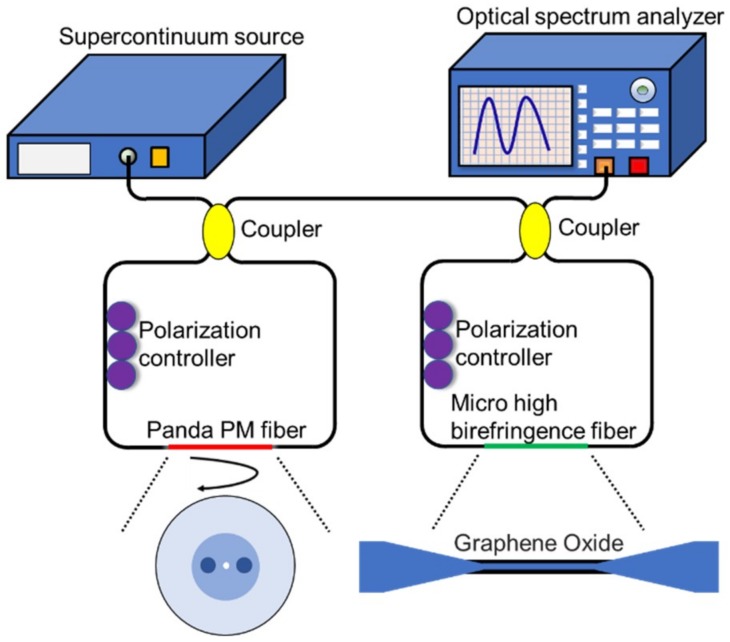
The schematic of the sensing system proposed based on VE.

The sensing system designed is shown in Figure 1. It includes two Sagnac loops, consisting of traditional two different high-birefringent fibers and 3 dB single-mode fiber couplers. One of the two interference rings consisted of a panda polarization-maintaining fiber as a filter, and the other loop with high-birefringent microfiber coated Graphene oxide was employed as a sensing channel. The sensitivity of the sensor can be continuously increased by reducing the size of the microfibers. Considering the mechanical properties and sensitivity of high-birefringent microfibers, we used the microfiber with a diameter of 6 μm. Both of the Sagnac loops have their own interference spectra, and the two interference spectra areas are superimposed to form a spectrum with a Vernier effect. When the RI of the surrounding solution varies, the spectrum of the sensor loop shifts, but the spectrum of the filter loop where the panda polarization-maintaining fiber is does not shift. Thus, we measure the analyte RI by the wavelength shift of the spectrum. The spectrum is superposed by the filter loop and the sensor loop. Compared to a single Sagnac loop sensor, the cascaded loops can output a spectrum with a Vernier effect that improves sensor sensitivity and reduces detection limits [14].

According to the Jones matrix [17], the transmission characteristic of a single Sagnac loop with a high-birefringent microfiber is expressed as:

(1) $$T=\sin^{2}\theta (1+\cos\frac{2\pi \Delta nL}{\lambda })$$

where θ=2πΔnL/λ is the phase shift between two polarization modes; Δn is the RI difference between the slow-axis and fast-axis, which indicates the birefringence of the Hi-Bi microfiber; *L* is the length of the Hi-Bi microfiber; and λ is the light wavelength. The transmission spectrum of the two cascaded Sagnac loops is multiplied by the transmittance of each of the two Sagnac loops, which can be written as:

(2) $$T=\sin^{2}\theta_{1}\sin^{2}\theta_{2}(1+\cos\frac{2\pi \Delta n_{1}L_{1}}{\lambda })(1+\cos\frac{2\pi \Delta n_{2}L_{2}}{\lambda })$$

Since $\theta_{1}$, $\theta_{2}$ are fixed values in the experiment and only have some influence on the amplitude of the transmission spectrum, they do not affect the spectral characteristics of the main research in this paper, so they are ignored and mainly study the influence of the parameters of the two fibers on the spectrum. Therefore, the transmission spectrum expression is:

(3) $$T=(1+\cos\frac{2\pi \Delta n_{1}L_{1}}{\lambda })(1+\cos\frac{2\pi \Delta n_{2}L_{2}}{\lambda })$$

The free spectrum range (FSR) of the transmission can be expressed as:

(4) $$FRS=\frac{\lambda^{2}}{\Delta nL}$$

The magnification of the cascaded Sagnac ring sensor can be expressed as [11]:

(5) $$M=\frac{FRS_{filter}}{|FSR_{sensor}-FSR_{filter}|}$$

where $FSR_{filter}$, $FSR_{sensor}$ represent the free spectral range of the filter loop and sensor loop, respectively.

**Figure 2:**
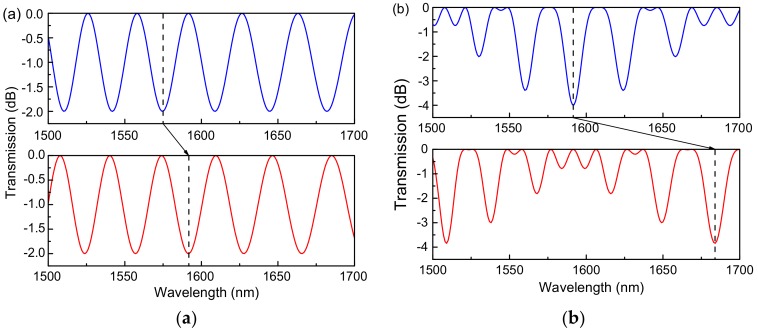
The simulated transmission spectrum shifts of (**a**) a single Sagnac-interferometer microfiber sensor and (**b**) a cascaded Sagnac-interferometer microfiber sensor.

As shown in Figure 2, the transmission spectra of the single Sagnac-interferometer sensor and cascaded Sagnac-interferometer sensor are simulated. $L_{1}$ = 30 cm is the length of the panda polarization-maintaining fiber, $L_{2}$ = 25 cm is the length of the Hi-Bi microfiber, and $\Delta n_{2}$ = 3 × 10^−4^ is the effective refractive index difference of the Panda polarization-maintaining fiber. When the numerical changes of analytes RI result in the effective index difference change to 3.1 × 10^−4^, the wave trough in the transmission spectra of the sensor consists of a single Sagnac loop shift 17 nm and that of cascaded Sagnac loop shift 92 nm. The results show that the sensitivity of the cascade Sagnac loop is 5.4 times that of the single loop.

## 3. Experience Results and Discussion

Figure 1 provides the schematic diagram of the experimental setup. The spectrometer used in the experiment was AQ6370D(YOKOGAWA, Tokyo, Japan), which has a measurement wavelength range of 600 to 1700 nm and a wavelength resolution of 0.02 nm. The light source used provides stable light waves with wavelengths from 1520 nm to 1620 nm. The polarization controller is a manual polarization controller, and the polarization state of the light can be changed by adjusting the angles of the three wave plates. We conducted the experiments in a temperature-controlled lab and the flow cell which was filled with different solutions was fixed onto an optical table to avoid any influence of temperature or strain.

### 3.1. Refractive Index Sensing

To assess the performance of the proposed sensor to measure analyte RI, six concentrations of NaCl solutions with the RI ranging from 1.3320 to 1.3414 measured by Abbe refractometer WYA-2S (INESA, Shanghai, China) are employed in the experiments. In the experiment, the length of PMF was 20 cm and that of the Hi-Bi microfiber was 25 cm, which were utilized in the sensing system. Considering both the mechanical properties and sensitivity of fiber optic sensors, the microfiber with the diameter of 6 μm was utilized to conduct experiments. All experiments were conducted in a lab, controlling temperature. In order to avoid the influence of temperature or strain, the flow cell was immobilized onto an optical table.

**Figure 3:**
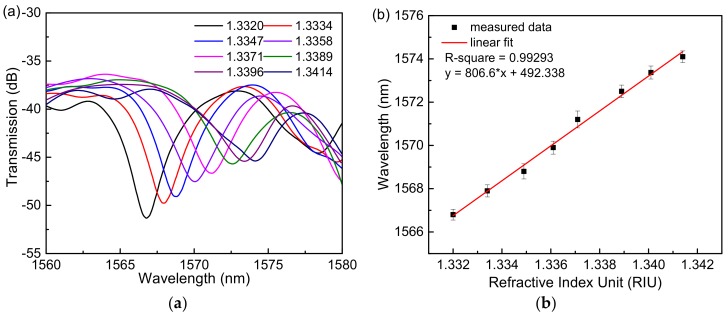
The experiment of the sensor based on a single Sagnac loop to measure analyte RI. (**a**) The transmission spectra in different analyte RI solutions. (**b**) Linear fitting curve.

**Figure 4:**
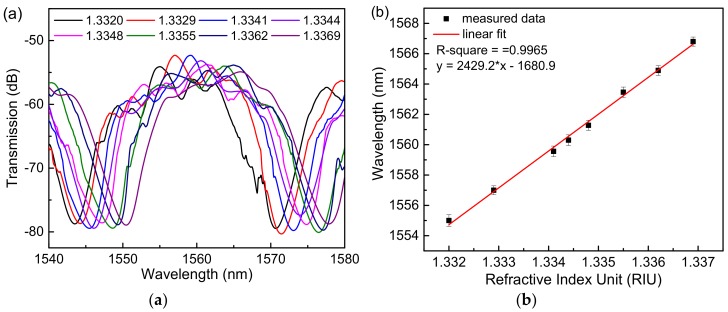
The experiment of the sensor based on a cascaded Sagnac-interferometer loop with the Hi-Bi microfiber to measure analyte RI. (**a**) The transmission spectra in different RI solutions. (**b**) Linear fitting curve.

The transmission spectra of the sensor based on a single Sagnac loop for different RI of NaCl solutions are shown in Figure 3a, and the linear fitting curves of the sensor based on a single Sagnac loop of the dips wavelength shift in response to the RI change are shown in Figure 3b. The sensitivity could reach 806.6 nm/RIU when the analyte RI varied from 1.3320 to 1.3414. By contrast, the transmission spectra of the cascade Sagnac-interferometer sensor based on VE are shown in Figure 4a. It is obvious that the cascading loop experiment produces a sensitizing effect, which has a certain relationship with the structure of the microfiber. When the spectrum of the output of the two loops is ideally superimposed in a certain band, the spectrum with amplification is the output, resulting in a certain VE, which in turn increases the sensitivity of the sensor. The results show that the sensitivity of the sensor can reach 2429.2 nm/RIU when the analyte RI varies from 1.3320 to 1.3369, which is about three times that of the single Sagnac loop. The experimental results are basically in accordance with the simulation data.

### 3.2. Biosensing

**Figure 5:**
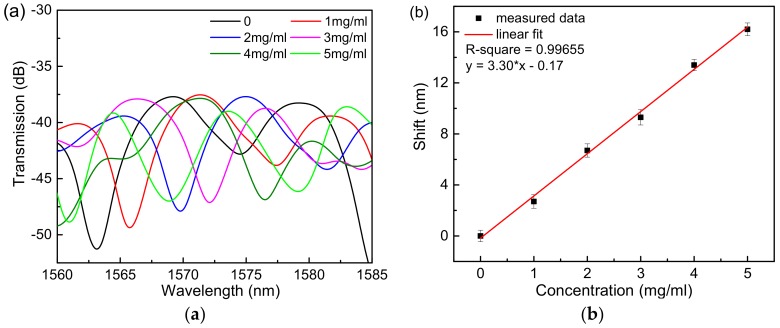
The experiment of a single Sagnac-interferometer loop to measure BSA solutions. (**a**) The transmission spectra. (**b**) Linear fit curve.

**Figure 6:**
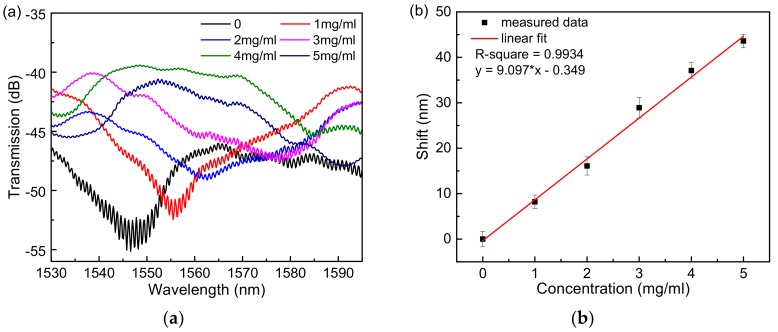
The experiment of the cascaded Sagnac-interferometer loop to measure BSA solutions. (**a**) The transmission spectra. (**b**) Linear fit curve.

The Hi-Bi microfiber was immersed in a 1 mol/L KOH solution to make the surface of the fiber hydrophilic and produce a hydroxyl group. The alkali-treated fiber was immersed in an APTES anhydrous ethanol solution with a mass fraction of 5% to silanize the surface, and the surface of the fiber was dropped with graphene oxide dispersion at a concentration of 80 μg/mL, followed by an oven at 70 °C. Heating for 30 min allowed GO to be reinforced. After the above steps, the surface of the Hi-Bi microfiber is fixed with a layer of suitable graphene oxide molecules, which has a good biocompatibility and large specific surface area [18,19]. The fiber is connected to the cascaded Sagnac sensor system to form the fiber biosensor studied in this paper. Bovine serum albumin solutions are prepared at concentrations of 0 mg/mL, 1.0 mg/mL, 2.0 mg/mL, 3.0 mg/mL, 4.0 mg/mL, and 5.0 mg/mL, respectively. Note that each time the bovine serum albumin solution is added to the fiber, it is washed with deionized water. The fiber ensures that there is no residual protein solution on the surface of the fiber. Since GO needs to adsorb BSA molecules, after the solution is added to the surface of the fiber, it is necessary to wait for about 1 min to stabilize the spectrum on the spectrometer and then save the data. By contrast, we first conducted the experiments of a single Sagnac loop sensor to determine the concentrations of BSA solution. The results are shown in Figure 5a, where it can be seen that the sensitivity can reach 3.30 nm/(mg×mL^−1^), and the linear fit shown in Figure 5b can be expressed as y = 3.30x − 0.17. Furthermore, we conducted the experiment of the cascaded Sagnac-interferometer loop to measure BSA solutions. It can be seen from Figure 6a that as the concentration of bovine serum albumin solution increases, the spectrum shifts to the long wavelength band. As the GO film adsorbs the BSA molecules, the RI of the sensor surface increases, which in turn causes the dips to move toward longer wavelengths. In Figure 6b, we determine the concentration of the BSA solution at concentrations of 0 mg/mL, 1.0 mg/mL, 2.0 mg/mL, 3.0 mg/mL, 4.0 mg/mL, and 5.0 mg/mL, respectively. The results show that the sensitivity can reach 9.097 nm/(mg×mL^−1^), which is approximately three times that of the single Sagnac loop, and the linear fit curve can be expressed as: y = 9.097x – 0.349. Regarding our experiment of detecting the concentration of BSA molecules, there is no need to manufacture the activated sensing film because of the interaction between pi-stacking of GO and BSA molecules [20].

## 4. Conclusions

A high-sensitive Sagnac-interferometer biosensor based on VE with a high-birefringence microfiber is proposed. The sensitivity enhancement is achieved by utilizing two cascade Sagnac interferometers. One of the two interference loops consists of a panda polarization-maintaining fiber as a filter, and the other is comprised of high-birefringent microfiber coated GO as a sensing channel. We theoretically analyzed the sensitivity of the sensor and verified it with experiments. The results show that the refractive index sensitivity reaches 2429 nm/RIU. The results show that the sensitivity of the cascade Sagnac loop is 5.4 times that of the single loop. Moreover, GO is employed to adsorb BSA molecules in order to achieve the desired detection results, which has a good biocompatibility and large specific surface area. The sensitivity to detect BSA can reach 9.097 nm/(mg×mL^−1^)
